# Follow‐up MRI appearance of the surgical site in dogs treated for thoracolumbar intervertebral disc herniation and showing ongoing or recurrent neurological symptoms

**DOI:** 10.1111/vru.13143

**Published:** 2022-08-12

**Authors:** Anne‐Lorraine Peschard, Paul Freeman, Marie‐Aude Genain

**Affiliations:** ^1^ Department of Veterinary Medicine The Queen's Veterinary School Hospital (QVSH) Madingley Road Cambridgeshire CB3 0ES UK

**Keywords:** canine, extrusion, hemilaminectomy, imaging, postoperative

## Abstract

Reherniation and reoperation rates of 4.5%–36% are reported in canine patients treated for intervertebral disc herniation (IVDH). Decision‐making for surgical reintervention can prove challenging, especially since common postoperative changes are poorly described on MRI. The purpose of this single‐center, retrospective, descriptive study was to describe the MRI characteristics of the surgical site in dogs treated for thoracolumbar IVDH and presenting for ongoing or recurrent neurological signs. Twenty‐one patients were included for a total of 42 MRI studies. Chondrodystrophic breeds, specifically Dachshunds, were overrepresented. Mean number of days between surgery and second MRI was 335 (range 2–1367). Metallic susceptibility artifacts were seen in seven of 21 cases (33%), but these were limited in extent, spanning on average 1.3 vertebral bodies. In 11 cases, spinal cord compression suspected to be clinically significant was found at the surgical site; the extradural compressive material consisted of intervertebral disc material only, or a combination of intervertebral disc material and hematoma or inflammatory changes in 10 cases, and a displaced articular process and fibrous tissue in one case. The latter is a newly described complication of mini‐hemilaminectomies. Paravertebral soft tissue changes and vertebral new bone formation varied according to the postoperative stage at which the patients were imaged. The results of this study supported the use of MRI as a diagnostic modality for spinal imaging following IVDH surgery, and showed that the presence of extradural disc material at a spinal surgical site is common along with various vertebral and paravertebral changes.

**Abbreviations**
ECVN: European College of Veterinary Neurology
IVD: intervertebral disc
IVDH: intervertebral disc herniation
IVDM: intervertebral disc material

## 1 INTRODUCTION

Recurrence of clinical signs affects an estimated 4.5–12.3%^1^, ^2^ or 6.4–36%^3^, ^4^, ^5^ of canine patients operated for intervertebral disc herniation (IVDH), with or without concurrent disc fenestration respectively. In such patients, decision‐making for surgical reintervention is made difficult by the lack of characterization of common postoperative changes on magnetic resonance imaging (MRI). The choice of imaging modality itself may be subject to query, due to concerns regarding MRI‐specific artifacts following surgery.

Residual spinal cord compression is commonly reported following surgery, with variable rates depending on the technique performed.^6^, ^7^, ^8^, ^9^, ^10^ When neurological signs have persisted or recurred postoperatively, the most commonly identified causes include herniation of additional intervertebral disc material (IVDM) from the surgical site, incomplete removal of the herniated IVDM, and formation of hematoma, seroma, or fibrous scar tissue.^10^, ^11^ However, deciding on surgical reintervention is complicated by the fact that the location and amount of extradural material do not always correlate with the severity of the reported clinical signs,^12^, ^13^ and additionally spontaneous regression of extradural IVDM has been reported in one dog.^14^ Therefore, reoperation may not always be indicated in patients showing ongoing pathological changes of the spinal cord and extradural space, and decision‐making would be more accurate if clinically significant or insignificant postsurgical findings were better characterized. This however poses the challenge of performing a longer general anesthetic and postoperative MRI study in patients that may not clinically benefit from it, and it may therefore be difficult to justify this in a clinical setting. Finally, although MRI is commonly accepted as the modality of choice for imaging the central nervous system, there is a risk of susceptibility artifacts rendering an MRI study non‐diagnostic following spinal surgery due to the presence of microscopic paramagnetic particles left from drilling bone, with one study speculating this risk to be high on low‐field MRI in postoperative patients.^15^ Susceptibility artifacts in MRI studies are visualized as a focal distortion of the image in proximity to adjacent materials of different magnetic susceptibility.^16^ They are greatest on gradient echo sequences, and will be larger with increasing magnetic field strength.^16^ In postoperative patients, these artifacts have been reported to be primarily caused by ferromagnetic objects, and this includes not only implants but also microscopic particles that can result from the use of a drill or rongeurs to obtain bony windows.^15^, ^17^, ^18^, ^19^

The objectives of this study were therefore to search for a population of dogs having undergone surgery for thoracolumbar IVDH as well as a postoperative MRI, and (1) assess the frequency and diagnostic impact of susceptibility artifacts on postoperative MRI studies; and (2) describe the changes seen at the surgical site on MRIs performed to investigate ongoing or recurrent neurological signs.

## 2 MATERIALS AND METHODS

### 2.1 Selection and description of subjects

This was a single‐center, retrospective, descriptive study. Records of canine patients seen at the Queen's Veterinary School Hospital (QVSH) between January 2009 and September 2020 were screened. Informed consent was obtained from all owners at the time of admission, and the authors obtained ethical approval from the Cambridge University Ethics & Welfare Committee reference CR553.

Criteria for case inclusion were (1) a diagnosis of thoracolumbar IVDH based on MRI findings and surgical confirmation; (2) a postoperative follow‐up MRI study was performed due to clinical need (slow recovery, incomplete recovery, or relapse of clinical signs after full recovery); and (3) all MRI studies included at least T2‐weighted (T2W) and T1‐weighted (T1W) sagittal and transverse sequences of the surgical site. A radiology resident (A.‐L.P.) and a European College of Veterinary Diagnostic Imaging (ECVDI)‐certified veterinary radiologist (M.‐A.G.) were responsible for final decisions for case inclusion or exclusion, as well as for consensus reviewal of the images.

### 2.2 Data recording and analysis

Patients’ records were reviewed by a veterinary radiology resident (A.‐L.P.). The following data were recorded: breed (classified as chondrodystrophic or non‐chondrodystrophic according to available lists of breeds associated with the FGF4‐12 and FGF4‐18 mutations^20^, ^21^), age, sex, neurological signs, and duration of onset at presentation and re‐presentation, number of days between first MRI, second MRI, and surgery, surgical technique, degree of recovery following treatment (full recovery being the absence of pain or neurological deficits at recheck either by the referring veterinarian or by the neurology team at the QVSH, and partial recovery being the persistence of neurological deficits beyond the expected time frame), treatment course following second MRI and finally patient follow‐up when available.

**FIGURE 1:**
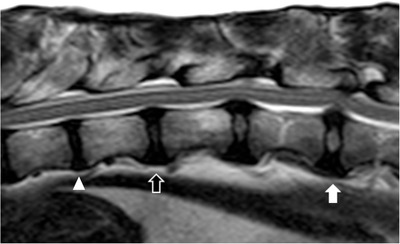
T2W mid‐sagittal image of the thoracolumbar junction of patient 9. This shows an example of a non‐degenerate IVD with an oval‐shaped T2W hyperintense nucleus pulposus (white arrow), a partially degenerate IVD with near complete loss of the T2W hyperintensity of the nucleus pulposus (empty arrow), and a completely degenerate IVD with complete loss of the T2W hyperintensity of the nucleus pulposus (arrowhead). Acquisition parameters: TR 2600 ms; TE 120 ms; slice thickness 3 mm; slice gap 3 mm; 1.5T MRI

The magnetic resonance (MR) images were retrieved and reviewed by the veterinary radiology resident (A.‐L. P.) and an ECVDI‐certified veterinary radiologist (M.‐A. G), using an open source 64‐bit medical image viewer (Horos version 3.3.6, Purview, Geneva, Switzerland; iMac Retina 4K, Apple Inc., California, United States). At the time of image review, the two assessors were blinded to patient data and previous imaging reports. The MR images were assessed for susceptibility artifacts compatible with the presence of metallic material (referred to as susceptibility artifacts for the remainder of the study), presence and characteristics of extradural material, and changes to the vertebrae, spinal cord, and paravertebral soft tissues. On the first MRI study of each patient, the degree of degeneration of the herniated IVD was classified as completely degenerate, partially degenerate, or non‐degenerate, based on an entirely lost, decreased, or intact T2W hyperintensity of the nucleus pulposus, respectively, and this is illustrated in Figure 1. Susceptibility artifacts were classified as absent, moderate, or marked, these last two categories depending on whether they allowed partial or no assessment of the adjacent vertebral canal, respectively. As susceptibility artifacts hindered the assessment of certain imaging criteria, this was taken into account when calculating prevalence rates of other imaging findings. The length of the cord affected by the susceptibility artifact was estimated in a number of vertebral bodies included in the area of signal distortion, using the T1W and T2W sagittal and transverse sequences. When present, extradural material was classified as markedly, moderately, mildly, or not compressive if it was occupying over 50% of the diameter of the vertebral canal, between 50 and 25%, less than 25%, or associated with an unchanged shape of the spinal cord and intact CSF column, respectively. The bony defects were assessed subjectively as “clearly visible” or “poorly visible”, and new bone formation was classified as “absent” or “present”. Tethering of the spinal cord was defined as the deviation of the spinal cord within the vertebral canal in the absence of extradural material, associated with focal loss of the extradural fat and CSF column between the spinal cord and the vertebra. The spinal cord was also assessed for the presence of focal ill‐defined T2W hyperintense areas that remained T1W isointense, estimated in vertebral body length. The observed changes were then reconsidered in light of the postoperative stage of the patient: the early postoperative period encompassed all patients imaged between 0 to 14 days post‐surgery (day 0 being the day of surgery), and the late postoperative period was from 14 days onwards.

## 3 RESULTS

### 3.1 Case demographics

Clinical and imaging details for each case are provided in Supporting Information 1. Over the study period, an approximate number of 660 dogs underwent thoracolumbar spine MRI and surgery. In total, 21 dogs (21/660, 3%) met the inclusion criteria of which 19(90%%) belonged to chondrodystrophic breeds (11 Dachshunds and Dachshund crosses, 3 Jack Russell Terriers, 1 Basset Hound, 1 Pug, 1 Lhasa Apso, 1 Springer Spaniel, and 1 French Bulldog) and 2(10%) belonged to non‐chondrodystrophic breeds (1 Dalmatian and 1 Crossbreed). Patient age ranged between 3 and 11 years 4 months old (median 6 years 2 months old). There were five entire female, six neutered female, two entire male, and eight neutered male dogs.

### 3.2 Initial diagnosis and clinical management

Duration of neurological clinical signs prior to first presentation ranged between 24 h and over 180 days (median 6 days). The most common clinical signs included back pain, lethargy, reluctance to walk, paraparesis, paraplegia, and ataxia.

The affected spinal segment was the thoracolumbar junction (T10‐L3) in 18 patients (18/21, 86%), and the lumbar spine (L3‐L7) in 3 patients (3/21, 14%).

All patients underwent spinal surgery within 24 hours of their first MRI. In total, 14 (14/21, 67%) dogs underwent a hemilaminectomy and seven (7/21, 33%) dogs undervent a mini‐hemilaminectomy. All surgeries were carried out by a European College of Veterinary Neurology (ECVN) resident, under the supervision of an ECVN‐boarded specialist. Several residents and a total of 10 ECVN specialists were involved, in many different combinations. Although the surgical approaches were grossly standardized for both hemilaminectomies and mini‐hemilaminectomies, technical specifications such as the number of muscle detachments and extent of surgical window in the bone were not available from the surgical reports. None of the surgeons involved used subcutaneous fat grafts. To create the vertebral bony window, all surgeons used a high‐speed drill (Hall^®^ Surgairtome Two^®^ and OralMax™ Pneumatic System, ConMed, Utica, New York) and rongeurs.

### 3.3 Technical parameters for the included MRI studies

In total, 42 MRI studies were available for review. All MRI studies but one were performed using a 0.27 Tesla permanent MR magnet (Esaote VetMR Grande, Genova, Italy). One postoperative MRI was performed using a 1.5 Tesla MR magnet (Phillips Achieva, Phillips Healthcare, Best, Netherlands). Besides the required T2W and T1W sagittal and transverse sequences focused on the surgical site, additional sequences were also reviewed when available including T1W dorsal, three‐dimensional hybrid contrast enhancement, and dorsal Short Tau Inversion Recovery. The sequences and settings varied between patients (Supporting Information 2).

### 3.4 MRI characteristics of the IVDH at first presentation

Pertinent imaging findings of sampled dogs are summarized in Supporting Information 3. Based on MRI findings and surgical confirmation, 16 of the chondrodystrophic dogs had an extrusion, two had a protrusion, and one had a protrusion associated with an extrusion while the two non‐chondrodystrophic dogs had an extrusion 1. Extradural hemorrhage associated with herniated IVDM was suspected in seven of 21 cases (33%), all of which were surgically confirmed. The IVDH was markedly compressive in eight cases (8/21, 38%), moderately compressive in four cases (4/21, 19%), and mildly compressive in nine cases (9/21, 43%). The herniated IVD was completely degenerate in 10 patients (10/21, 48%), partially degenerate in 10 patients (10/21, 48%), and nondegenerate in one patient (1/21, 5%).

### 3.5 Second MRI examination

The mean number of days between surgery and second MRI study was 335 (range 2–1367 days) days. Eight patients were included in the early postoperative period (range 2–14 days, mean 6 days), and 13 patients in the late postoperative period (range 52–1367 days, mean 537 days).

### 3.6 Susceptibility artifacts

**FIGURE 2:**
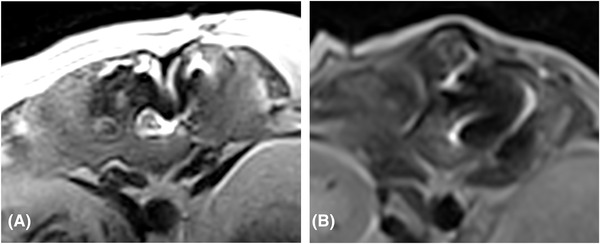
T2W transverse images of patient 19 (A) and patient 16 (B), showing a moderate and marked susceptibility artifact, respectively. The susceptibility artifact is visible in both cases as an area of distorted signal, to the left lateral aspect of the vertebral canal. Acquisition parameters: TR 6140 (A and B) ms; TE 120 (A and B) ms; slice thickness 3 mm; slice gap 3.5 mm; 0.25T MRI

Susceptibility artifacts were present in seven cases (7/21, 33%), of which three (3/21, 14%) cases were classified as moderate and four (4/21, 19%) cases as marked. An example of each magnitude of signal distortion is available in Figure 2. The area of signal distortion spanned on average 1.33 vertebral bodies (range 0.5–2), with no difference in extent between T2W and T1W sequences. All four marked susceptibility artifacts were associated with mini‐hemilaminectomies, and the three moderate ones with hemilaminectomies. These artifacts were present in 25% (2/8) and 38% (5/13) of studies performed in the early and late postoperative periods respectively, and were not present on the single high‐field study.

Due to interference from susceptibility artifact at the surgical site, certain patients had to be excluded from the assessed population for the following imaging criteria located at the surgical site: degree of compression of the spinal cord, length of intramedullary T2W hyperintensity, tethering of the spinal cord, bony changes, reduction in volume of the epaxial muscles, change in intensity of the ipsilateral epaxial muscles, and presence of collections of fluid in the paravertebral soft tissues. In some patients with moderate susceptibility artifact, the disruption in signal was lateralized enough to assess the adjacent vertebral canal accurately, and this was taken into account when excluding cases for certain imaging features. Eventually, exclusions from specific imaging features applied to one patient from the early postoperative group (case 20) and four patients from the late postoperative group (cases 2, 10, 11, 16). Purely imaging‐based results are therefore presented out of a total of 7 patients for the early postoperative group, out of 9 patients for the late postoperative group, and out of a total of 16 when both groups are combined.

### 3.7 Clinical outcomes following initial surgery and cause of spinal compression on second MRI

Among the eight patients of the early postoperative group, only one (1/8, 13%) patient recovered well from the surgery before relapsing (case 13), with the others showing poor post‐surgical improvement or even deterioration of their neurological signs. Five (5/8, 63%) of these patients had evidence of ongoing or recurrent compression of the spinal cord at the surgical site (by extradural disc material and hemorrhage, hematoma, or inflammatory tissue), including case 13, with only one (1/8, 13%) patient having neurological signs attributed to a disc extrusion distant from the surgical site, and one (1/8, 13%) patient showing no evidence of spinal cord compression. In one (1/8, 13%) patient, the MRI was inconclusive due to susceptibility artifact and no second surgery was performed, precluding an accurate diagnosis. In this patient, the surgical site was presumed to be the site of the focal myelopathy responsible for the neurological signs in the absence of other visible lesions in the remainder of the studied spinal segment. In the 5 patients with identified compressive extradural material at the surgical site, this was causing marked, moderate or mild compression of the spinal cord in one, three, and one patients, respectively. Four patients from this group underwent surgical revision of the surgical site and all but one showed improvement of their neurological status after the second surgery.

Of the 13 patients that belonged to the late postoperative group, four patients had a poorly diagnostic MRI study at the level of the surgical site due to susceptibility artifact. In this group, 10 (10/13, 77%) patients made a full recovery following surgery and three (3/13, 23%) patients only made a partial recovery. These three patients showed mild chronic neurological deficits that eventually acutely deteriorated; one had no evidence of ongoing compression at the surgical site and a compressive distant IVDH, 1 had evidence of mild compression at the surgical site only, and one had a compressive distant IVDH with a poorly characterizable surgical site due to localized susceptibility artifact. Of the 10 patients that had recovered fully from the surgery, three had imaging evidence that compression of the spinal cord at the surgical site was the cause of the presenting neurological signs and an additional patient had suspected spinal cord compression at the surgical site in the absence of other visible compressive lesions of this spinal segment. This last patient (case 16) underwent surgical revision of the previous mini‐hemilaminectomy site which resulted in removal of a displaced articular facet and fibrous material from the vertebral canal. Of the three patients that recovered fully from initial surgery, then represented in the late postoperative period with clinically significant spinal cord compression at the surgical site by extradural IVDM (cases 1, 6, 8), two had concurrent IVD fenestration at the time of initial surgery. Finally, six patients were thought to have relapsed due to IVDH at a different disc space, of which two were free of compression at the surgical site, two showed evidence of extradural compression at both the surgical site and a distant disc space, and two had too much susceptibility artifact to characterize the surgical site fully on MRI. The degree of spinal cord compression at the surgical site was deemed to be moderate in one patient, and mild in five patients. Five patients underwent spinal surgery to remove extradural material at a site distant from the previous surgical site, with only one patient undergoing revision of its previous surgical site (case 16).

When assessing specifically the central T2W hyperintensity of the intervertebral discs that were not herniated on the first MRI study but herniated on the second study, six were classified as completely degenerate, two as partially degenerate, and one as nondegenerate on the initial MRI.

### 3.8 Changes to the vertebrae

The changes to the vertebrae could not be accurately described in the presence of adjacent susceptibility artifact, and therefore the aforementioned 5 postoperative MRI studies were excluded from the following results.

**FIGURE 3:**
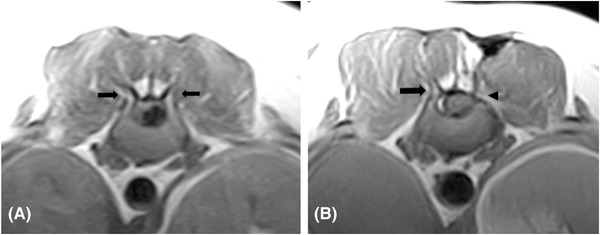
T1W transverse images of the same patient (case 8), preoperative (A), and 839 days post hemilaminectomy (B). The arrows point to the normal articular processes, and the arrowhead points to the postoperative defect in the left side of the lamina and absent left articular process. Acquisition parameters: TR 650 (A) and 890 (B) ms; TE 26 (A and B) ms; slice thickness 4 (A and B) mm; slice gap 4 (A and B) mm; 0.25T MRI

Bony defects were clearly visible without evidence of new bone formation in 10 patients (10/16, 63%); this included all patients (7/7, 100%) from the early postoperative period, and three patients (3/9, 33%) from the late postoperative period, up to 839 days after surgery (Figure 3).

Bony defects were poorly visible without clear evidence of bony hyperplasia in three of 16 patients (19%), and hyperplastic vertebral new bone formation was identified in three of 16 patients (19%). These six patients all belonged to the late postoperative group.

**FIGURE 4:**
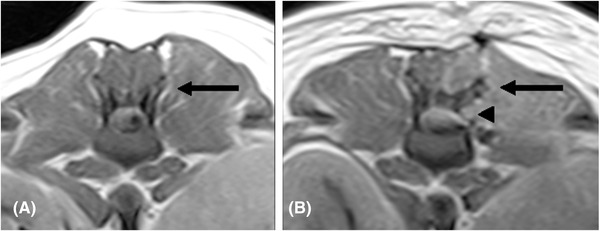
T1W transverse images of the same patient (case 17), pre‐operative (A), and postoperative (B). The arrow points to the ventrally displaced left cranial articular process following left‐sided mini‐hemilaminectomy. A small bony defect in the left lamina of the vertebra is visible ventral to the affected articular process (arrowhead). Acquisition parameters: TR 950 (A and B) ms; TE 26 (A and B) ms; slice thickness 4 mm; slice gap 4 mm; 0.25T MRI

There was evidence of displacement of a cranial articular facet of L1 following a left‐sided T13‐L1 mini‐hemilaminectomy in two patients imaged within a week of surgery. One of them showed a clearly visible non‐compressive displaced articular facet on MRI (Figure 4), while the MRI study of the other patient was poorly diagnostic due to susceptibility artifact but a displaced compressive articular facet was surgically confirmed.

### 3.9 Changes to the spinal cord

On 13 of the first MRI studies (13/21, 62%), the spinal cord showed a focal area of T2W hyperintensity that remained T1W isointense, overlying the diseased IVD space. This spanned a length of less than two vertebral bodies in nine cases (43%) and over two vertebral bodies in four cases (19%). Of these four cases, two made a full recovery after the initial surgery, one remained mildly ataxic for 27 months postsurgery, and one developed myelomalacia within days of surgery, highly suspected from the clinical evolution and second MRI and confirmed at postmortem examination.

On the second MRI studies, after exclusion of the five cases with susceptibility artifacts, this focal T2W hyperintense region of the spinal cord had increased in length (7/16 cases, 44%), decreased in length (6/16 cases, 38%), or remained absent (3/16 cases, 19%).

The increase in length was superior to two vertebral bodies in three cases (all of which had ongoing or worsening neurological signs following surgery, and belonged to the early postoperative group), equivalent to 1 to 2 vertebral bodies in length in two cases (both of which had a slow recovery and acutely deteriorated following initial surgery, requiring repeat imaging during the early postoperative period and surgical reintervention), or inferior to one vertebral body in two cases (both made a full recovery after the initial surgery and were imaged in the late postoperative period).

Of the nine cases in which either the spinal cord was within normal limits, or the T2W hyperintensity of the spinal cord decreased, one belonged to the early postoperative group and eight to the late postoperative group. Five of these patients had a good clinical outcome, and four were lost to follow‐up.

**FIGURE 5:**
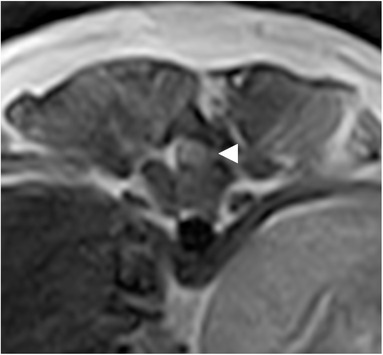
T2W transverse images of patient 18, showing deviation of the spinal cord toward the left‐sided hemilaminectomy site (arrowhead), in the absence of extradural material. The contralateral hyperintense CSF column is widened, while as it is interrupted on the side of the tethering. Also note the T2W hyperintense tissue along the left side of the spinous process, and the reduction in volume of the left epaxial muscles. Acquisition parameters: TR 5000 (A and B) ms; TE 120 (A and B) ms; slice thickness 3 mm; slice gap 3.5 mm; 0.25T MRI

Of the 16 follow‐up MRI studies that were not affected by susceptibility artifacts, tethering of the spinal cord to the surgical site was present in four (4/16, 25%) studies, all belonging to the late postoperative group. Concurrent attenuation of the CSF and fat columns ipsilateral to the tethering was visible, along with widening of the contralateral CSF column. This is illustrated in Figure 5. Two of these patients had a good neurological outcome, and 2 were lost to follow‐up.

### 3.10 Soft tissues

Unilateral changes in the paravertebral soft tissues were also appreciated in the 16 postoperative MRI studies that were not affected by susceptibility artifacts, both in the early (7 studies) and in the late (9 studies) postoperative groups.

Common changes seen in the patients imaged within 14 days of surgery included increased volume of the epaxial muscles over the surgical site, associated with a diffuse to patchy T2W hyperintensity that remained T1W isointense (7/7 cases, 100%), focal collections of T2W markedly hyperintense, T1W mildly hyperintense to muscle material in the paravertebral soft tissues (6/7 cases, 86%), and multifocal subcutaneous small rounded signal voids (5/8 cases, 63% ‐ the subcutaneous positioning of these signal voids allowed their visualization despite susceptibility artifacts). These changes were never seen in patients imaged later than 14 days after surgery.

From 14 days onward, five of nine (56%) patients showed a focal reduction in the volume of the epaxial muscles ipsilateral to the surgery, and these muscles had a similar increased signal intensity on both T2W and T1W sequences when compared to the preoperative studies. A tract of T2W and T1W hyperintense tissue could be identified in two (2/9, 22%) patients of this group along the spinous process, in the area of the surgical approach. This is also illustrated in Figure 5.

Finally, a common finding that could be appreciated in all 21 postoperative MRI studies despite the presence of susceptibility artifacts was the presence of thin linear T2W/T1W hypointense subcutaneous tracts in the area of the surgical approach, found in all patients in the early postoperative group (100%), and eight of 13 patients in the late postoperative group (62%).

## 4 DISCUSSION

Findings from this study supported using follow‐up MRI of dogs treated for IVDH for characterizing the changes at the surgical site, with only a few occurrences of MRI studies being rendered poorly diagnostic due to susceptibility artifacts. Several of these postoperative changes could also be related to the stage of the postoperative period, with some being more or less common depending on the time elapsed since surgery. Due to the presence of pain or neurological deficits in the patients included in this study, the clinical impact of these changes diagnosed on MRI could not be ascertained. The population included in the present study showed characteristics that were similar to those from other publications on canine IVDH, with chondrodystrophic breeds and more particularly Dachshunds being overrepresented, and most patients being middle‐aged (median age of 6 years 2 months old).^4^, ^22^, ^23^, ^24^, ^25^

In 16 of 21 patients (76%) included in this study, metallic susceptibility artifacts were either not present or did not hinder the evaluation of the vertebral canal. This does not support the findings of an earlier study, that speculated that susceptibility artifacts would be common postoperatively and therefore MRI may not be an adequate imaging modality for patients that had undergone spinal surgery.^15^ It is also interesting to note that when present, the susceptibility artifact would affect the visualization of the adjacent vertebral canal, but this was limited in length and therefore most studies impacted by susceptibility artifacts would remain diagnostic for most of the spinal cord in the field of view. Certain setting adjustments may also be performed to reduce the impact of susceptibility artifacts on the diagnostic quality of MRI sequences, but this was beyond the scope of the present study. Finally, it was also noted that the marked susceptibility artifacts were associated with mini‐hemilaminectomy procedures, whereas the moderate ones were all associated with hemilaminectomy procedures. Although the equipment used and burring intensity and process were similar for both surgical techniques, a possible reason for this might be better access and therefore more thorough intraoperative saline flushing of the surgical site in hemilaminectomies as compared to mini‐hemilaminectomies. However, the number of cases is low and this may therefore not be a significant reproducible finding.

Extradural IVDM at the surgical site was described in 11 patients (5 from the early postoperative group and 6 from the late postoperative group), which represents 52% of the study population. It remains unclear whether this was residual from surgery or newly herniated IVDM. The imaging evidence of persistent extradural compression of the spinal cord alongside the history of clinical deterioration or failure to improve prompted surgical reintervention in all patients which showed compression of the spinal cord at the surgical site during the early postoperative period.

The clinical significance of the presence of spinal compression at the surgical site was considered in light of the presence or absence of other spinal lesions. Compression at the surgical site was suspected to be the cause of the neurological signs in 11 of the included patients, in the absence of another site of significant spinal cord compression. Following conclusions from previous studies,^1^, ^2^, ^3^ these patients would have been expected to present early in the postoperative period, with incomplete recovery from surgery. Conversely, these 11 patients showed variable degrees of recovery, up to full recovery as assessed by the neurology team, and a wide range of postoperative periods, up to years after the initial spinal surgery. This shows that an initial full recovery and/or a long period of time elapsed since surgery does not preclude that the disc space involved in the original surgery may be again a site of spinal cord compression. Because of the initial good recovery, the authors speculate that the compressive extradural IVDM at the surgical site result from a recurrence of disc herniation rather than material left at the previous surgery, and this happened in 2/3 cases despite IVD fenestration at the time of decompressive surgery. In these 11 patients, the extradural causes for spinal cord compression included ones commonly reported, such as IVDM, hemorrhage, and inflammatory changes, but interestingly in one case, the compression was caused by a displaced articular process. The latter was one of two cases that suffered from the displacement of a cranial articular process of L1 following a mini‐hemilaminectomy T13‐L1. Although there has been a report of spinal instability resulting from bilateral mini‐hemilaminectomy and pediculectomy in a dog,^26^ displacements of an articular process has not yet been described as a potential complication of mini‐hemilaminectomies. As evidenced by the present study, this may warrant surgical reintervention if the displaced articular process extends into the vertebral canal.

Nine patients showed a disc hernia distinct from the initial surgical site on their second MRI. When reviewing the central T2W hyperintensity of these intervertebral discs on the first MRI study of each patient, most (8/9, 89%) showed some degree of loss of this hyperintensity, indicating degeneration. This is consistent with the pathophysiology of IVD disease and previous publications.^5^, ^25^, ^27^

Bony defects were more clearly discerned in the early postoperative period, where all patients imaged within 2 weeks of surgery showed a clearly identifiable gap in the vertebral bone corresponding to the surgical window. Marked vertebral new bone formation was found in three MRI studies (out of 16 diagnostic postoperative studies, 19%), all in the late postoperative period. Interestingly, different degrees of bony defects and new bone formation could be seen in patients imaged at similar postoperative stages, showing that the amount of new bone formation did not necessarily relate to the amount of time elapsed since surgery. From a clinical point of view, the presence of a large amount of new bone formation may complicate the surgical approach if surgical reintervention is required—this was not the case for the included patients.

When considering the difference in length of the T2W hyperintense region of the spinal cord between the first and second MRI studies, the five patients in which it increased in length by more than one vertebral body had a poor clinical outcome, whereas the other seven patients for which follow‐up was available were associated with good outcomes. Hypotheses as to the cause of this hyperintensity include contusion, edema, and gliosis, depending on the time frame since the insult to the spinal cord occurred—the mechanism that causes this hyperintensity to increase or decrease in length over time is however unknown.

Tethering of the cord to the surgical site was found in a quarter of patients with postoperative MRIs free of susceptibility artifacts. All of these four patients belonged to the late postoperative group. It is unclear why some patients developed this feature compared to others, and it is also difficult to estimate the clinical impact of this as at least two of these patients had a good neurological outcome.

Unilateral changes in the epaxial muscles were common, and their nature varied depending on the time elapsed since surgery. In all patients imaged within 2 weeks of surgery, the epaxial muscles over the surgical site were mildly swollen, with a change in signal intensity consistent with inflammation or edema. Collections of fluid in the soft tissues adjacent to the surgical site, thought to be seroma or hemorrhagic fluid, were also common in the 2 weeks postsurgery (86% of cases). Finally, in five patients, multifocal small subcutaneous signal voids were present and thought to be due to subcutaneous emphysema and/or suture material. Soft tissue changes were evaluated subjectively and there was no appreciable difference between the patients that underwent hemilaminectomy and those that underwent mini‐hemilaminectomy.

After the initial postoperative two weeks, just over half of patients (56%) showed a reduced volume of the operated epaxial muscles compared to the contralateral musculature, occasionally associated with an increase in signal intensity compatible with fatty infiltration. Although no muscle biopsies were performed, possible explanations for this include loss of innervation and/or loss of blood supply, causing muscular atrophy.

The T2W and T1W hyperintense tract visible in two patients of the late postoperative group along the spinous process of the operated vertebra were hypothesized to be fatty infiltration in the area of dissection of the epaxial muscles from their bony attachments.

Finally, approximately three quarters of the included population (76%) showed a linear tract in the subcutaneous fatty tissues in the area of the surgical approach, of a signal intensity suggestive of fibrosis or scar tissue.

Limitations of this study include first of all its retrospective nature, which introduces variables such as non‐standardized MRI sequences, and record‐keeping. The authors however implemented strict inclusion criteria to mitigate this. Several different combinations of residents and specialists were involved in the surgeries, and although surgical approaches for both hemilaminectomies and mini‐hemilaminectomies are standardized to some degree, certain aspects of these techniques will inherently vary with the surgeon involved. Surgical reports did not offer details such as the length of the incision, the number of muscle detachments, and the size of the bony window. All the data were also collected from a single institution, and may therefore be biased by a number of factors such as the clinicians involved, the surgical material used, and the imaging acquisition methods. Case numbers were also small, although still comparable to other studies on the subject. Because several patients were treated conservatively following their second MRI, surgical confirmation was often not available for the findings of the follow‐up MRIs. All the patients included in the present study underwent a second MRI due to ongoing or relapsed neurological signs; it is therefore uncertain whether the changes described would be found in patients that recover uneventfully, and to what degree they contributed to the recurrence of neurological signs in our patients. Assessment of bony changes would also potentially have been more accurate on CT than MRI, and therefore further studies using CT to evaluate bony healing and remodeling following spinal surgery may be of interest. Finally, it is also important to consider that the low diagnostic impact of the susceptibility artifacts in this study may only be valid for low‐field MRI, as it is less sensitive to local magnetic field inhomogeneities compared to high‐field MRI.

In conclusion, findings from this sample of dogs indicated that, contrary to what was suggested in a previous study, metallic susceptibility artifacts are uncommon in postoperative low‐field MRI studies, and that the disruption caused to the diagnostic quality of the MR images is focally limited to the surgical site. MRI can therefore be considered an adequate modality for patients that require postsurgical spinal imaging, although further studies may be required to extrapolate this finding to high‐field magnets. Additionally, this study has also shown that the appearance of spinal surgical sites on MRI is variable, with certain findings increasing or decreasing in prevalence according to the stage of the postoperative period. Additionally, ongoing or recurrent compression of the spinal cord at the surgical site is common, even in patients that have made a full recovery and present with a late recurrence of clinical signs.

## LIST OF AUTHOR CONTRIBUTIONS

**Category 1**

(a) Conception and Design: Peschard, Freeman, Genain
(b) Acquisition of Data: Peschard
(c) Analysis and Interpretation of Data: Peschard, Freeman, Genain

**Category 2**

(a) Drafting the article: Peschard
(b) Revising Article for Intellectual Content: Peschard, Freeman, Genain

**Category 3**

(a) Final Approval of the Completed Article: Peschard, Freeman, Genain

**Category 4**

(a) Agreement to be accountable for all aspects of the work ensuring that questions related to the accuracy or integrity of any part of the work are appropriately investigated and resolved: Peschard, Freeman, Genain

## CONFLICT OF INTEREST

The authors have declared no conflict of interest.

## PREVIOUS PRESENTATION OR PUBLICATION DISCLOSURE

Preliminary results were presented at a QVSH meeting on Friday 5th of February 2021. The abstract was presented at the pre‐BSAVA EAVDI meeting on Wednesday 24th of March 2021.

## EQUATOR NETWORK DISCLOSURE

An EQUATOR network checklist was not used.

## Supporting information

Supplement 1: Details for individual cases.

Supplement 2: Technical parameters used for MRI studies (slice thickness, slice interval, repetition time [TR], echo time [TE], inversion recovery [IR], echo train length [ETL], and number of signals averaged [NSA]) and number of studies for each MRI sequence used.

Supplement 3: Frequencies of the main imaging findings for included dogs, according to the stage of the postoperative period at which the second MRI was performed.
