# Supplementary material for: Follow‐up MRI appearance of the surgical site in dogs treated for thoracolumbar intervertebral disc herniation and showing ongoing or recurrent neurological symptoms
Source: Vet Radiol Ultrasound. 2022 Aug 12;64(1):95–104. doi: 10.1111/vru.13143 (PMC10086782; doi:10.1111/vru.13143)
Supplement: Supplementary file 1 — Supplement 1: Details for individual cases. [file VRU-64-95-s003.docx]

| Case number | Breed | Age | Sex | Time lapsed between surgery and second MRI (in days) | Duration of clinical signs prior to initial presentation (in days) | Type of intervertebral disc herniation | Degree of compression on 1st MRI | Length of intramedullary T2W hyperintensity (in vertebral bodies) on 1st MRI | Surgical technique | Surgical site | Full recovery obtained following first surgery | Onset of clinical signs prior to 2nd presentation | Cause of cord compr ession at the surgical site on 2nd MRI | Degree of compression of the spinal cord at the surgical site | Length of intramedullary T2W hyperintensity (in vertebral bodies) associated with the surgical site | Tethering of the spinal cord | Bony changes | Reduction in volume of the epaxial muscles | Change in intensity of the ipsilateral epaxial muscles | T2W and T1W hypointense linear tract in the subcutaneous fat dorsal to the surgical site | Collection of fluid in the paravertebral soft tissues adjacent to the surgical site | Signal voids or susceptibility artefacts | Length of metallic susceptibility artefact (in vertebral bodies affected by the artefact) | Suspected cause of neurological signs based on 2nd MRI | 2nd treatment course | Outcome |
| --- | --- | --- | --- | --- | --- | --- | --- | --- | --- | --- | --- | --- | --- | --- | --- | --- | --- | --- | --- | --- | --- | --- | --- | --- | --- | --- |
| 1 | Dachshund | 5y | FE | 174 | 14 | Extrusion | Marked | 1.5 | Hemilaminectomy with fenestration | T13-L1 left-sided | Yes | Acute | Extradural IVDM | Moderate | 1 | N/A^1^ | Well-defined bony defect | Absent | None | Present | Absent | Subcutaneous small susceptibility artefacts | N/A | Ongoing or recurrent extradural IVDM at the surgical site | Conservative | Unknown |
| 2 | Dachshund | 5y8m | FE | 363 | 3 | Extrusion and haemorrhage | Moderate | 1.5 | Mini-hemilaminectomy with fenestration | T13-L2 left-sided | No | Peracute | Unable to assess due to metallic susceptibility artefact. | | | | | | | Absent | Unable to assess due to metallic susceptibility artefact. | Metallic susceptibility artefact – marked.  Rounded subcutaneous signal voids and small susceptibility artefacts. | 1 | IVD extrusion distant from the surgical site | Right-sided hemilaminectomy T12-T13. | Good |
| 3 | Dachshund cross | 4y | MN | 152 | 2 | Extrusion associated with haemorrhage | Moderate | 0 | Mini-hemilaminectomy with fenestration | T12-L1 right-sided | Yes | Peracute | No compression present | Absent | 0 | Present | Poorly defined bony defect, and bony hyperplasia | Present - ipsilateral to the surgical site | Focally T2W and T1W hyperintense | Present | Absent | Absent | N/A | IVD extrusion distant from the surgical site | Left sided hemilaminectomy L1-L2 with fenestrations | Good |
| 4 | Cross Breed | 8y | MN | 14 | 4 | Extrusion and protrusion | Mild | 1 | Hemilaminectomy with fenestration | T13-L1 left-sided | No | Acute | Extradural IVDM, haematoma and healing tissue | Moderate | 1.5 | N/A^1^ | Well-defined bony defect | Absent | Focally T2W hyperintense and T1W isointense | Present | Absent | Absent | N/A | Ongoing compression by extradural IVDM and haematoma / fibrous scar at the surgical site | Revision of surgical site | Good |
| 5 | Jack Russel Terrier | 6y | MN | 13 | 30 | Extrusion and haemorrhage | Mild | <1 | Hemilaminectomy with fenestration | L1-L2 right-sided | No | Peracute | No compression present | Absent | 3 | Absent | Well-defined bony defect | Absent | Focally T2W hyperintense and T1W isointense | Present | Present (large) | Rounded small subcutaneous signal voids | N/A | IVD extrusion distant from the surgical site | Conservative. | Fair |
| 6 | Springer spaniel | 9y | ME | 1367 | 16 | Protrusion | Moderate | 0 | Mini-hemilaminectomy | L1-L2 left-sided | Yes | Subacute | Extradural IVDM | Mild | <1 | N/A^1^ | Poorly defined bony defect, and bony hyperplasia. | Absent | None | Absent | Absent | Absent | N/A | Ongoing or recurrent extradural IVDM at the surgical site | Conservative | Unknown |
| 7 | Dachshund | 5y | FN | 2 | 2 | Extrusion | Marked | 0 | Hemilaminectomy with fenestration | T12-T13 right-sided | No | Peracute | Extradural IVDM and haemorrhage | Moderate | 1.5 | N/A^1^ | Well-defined bony defect | Absent | Focally T2W hyperintense and T1W isointense | Present | Present (large) | Rounded small subcutaneous signal voids | N/A | Ongoing or recurrent extradural IVDM at the surgical site | Revision of surgical site | Good. |
| 8 | Dachshund cross | 5y | FN | 839 | 10 | Extrusion | Marked | 2 | Hemilaminectomy with fenestration | T12-T13 right-sided | Yes | Acute | Extradural IVDM | Mild | 0 | N/A^1^ | Well-defined bony defect | Present - ipsilateral to the surgical site | Track of fatty tissue along the spinous process | Absent | Absent | Absent | N/A | Ongoing or recurrent extradural IVDM at the surgical site | Conservative. | Good. |
| 9 | Dalmatian | 10y | MN | 1230 | 7 | Extrusion and haemorrhage | Mild | 1 | Hemilaminectomy | L4-L5 Right-sided | Yes | Acute | No compression present | Absent | 0 | Present | Poorly defined bony defect. | Present - ipsilateral to the surgical site | None | Present | Absent | Absent | N/A | IVD extrusion distant from the surgical site | Conservative | Good |
| 10 | Dachshund | 4y | FN | 972 | 2 | Extrusion | Moderate | 0 | Mini-hemilaminectomy | T12-T13 left sided | Yes | Chronic | Unable to assess due to metallic susceptibility artefact. | | | | | | | Present | Unable to assess due to metallic susceptibility artefact. | Metallic susceptibility artefact - marked | 1 | IVD extrusion distant from the surgical site | Conservative. | Unknown |
| 11 | Dachshund | 6y | FN | 201 | 14 | Extrusion | Marked | <1 | Hemilaminectomy with fenestration | L2-L3 left-sided | Yes | Acute | Unable to assess due to metallic susceptibility artefact. | | | | | | | Present | Unable to assess due to metallic susceptibility artefact. | Metallic susceptibility artefact - moderate | 1.5 | IVD extrusion distant from the surgical site | Left-sided mini-hemilaminectomy T13-L1. | Good |
| 12 | Dachshund | 7y | MN | 819 | 7 | Extrusion and haemorrhage | Marked | 2 | Hemilaminectomy | T13-L1 right-sided | No | Subacute | No compression present | Absent | <1 | Absent | Poorly defined bony defect. | Absent | Focally T2W and T1W hyperintense | Present | Absent | Absent | N/A | IVD extrusion distant from the surgical site | T11-T12 hemilaminectomy and fenestrations. | Good |
| 13 | Pug | 11y4m | MN | 2 | 6 | Protrusion | Mild | <1 | Hemilaminectomy | T11-T12 right-sided | Yes | Acute | Extradural IVDM and inflammatory tissue | Moderate | 0 | N/A^1^ | Well-defined bony defect | Absent | Focally T2W hyperintense and T1W isointense | Present | Present (large) | Rounded small subcutaneous signal voids | N/A | Ongoing compression by extradural IVDM and haematoma at the surgical site | Conservative. | Good |
| 14 | Jack Russel Terrier | 4y | FE | 85 | 90 | Extrusion | Mild | 0 | Hemilaminectomy with fenestration | T13-L1 right sided | Yes | Chronic | Extradural IVDM | Mild | 0 | Present | Poorly defined bony defect. | Absent | None | Present | Absent | Rounded small subcutaneous signal voids | N/A | IVD extrusion and protrusion distant from the surgical site | Conservative. | Unknown |
| 15 | Dachshund | 6y8m | FN | 3 | 5 | Extrusion | Marked | 1 | Mini-hemilaminectomy with fenestration | T11-T12 left-sided | No | Acute | Extradural IVDM, and haemorrhage | Marked | 3 | N/A^1^ | Well-defined bony defect | Absent | Focally T2W hyperintense and T1W isointense | Present | Present (large) | Rounded small subcutaneous and paravertebral signal voids | N/A | Ongoing or recurrent extradural IVDM at the surgical site | Revision of surgical site | Good |
| 16 | Dachshund | 5y | ME | 139 | 21 | Extrusion and haemorrhage | Marked | 1.5 | Mini-hemilaminectomy | T13-L1 left-sided | Yes | Acute | Unable to assess due to metallic susceptibility artefact. | | | | | | | Present | Unable to assess due to metallic susceptibility artefact. | Metallic susceptibility artefact - marked | 2 | Presumed to be surgical site as no other lesions seen. | Revision of surgical site: articular facet and fibrous material removed. | Fair |
| 17 | Jack Russel Terrier | 3y | MN | 2 | 3 | Extrusion and haemorrhage | Marked | 0 | Mini-hemilaminectomy | T13-L1 left-sided | No | Peracute | Extradural IVDM, haematoma and healing tissue | Mild | 1.5 | N/A^1^ | Well-defined bony defect. Asymmetry of the cranial articular processes of L1, with ventrolateral displacement of the left cranial articular process. | Present - ipsilateral to the surgical site | Focally T2W hyperintense and T1W isointense | Present | Present (moderate and multifocal) | Metallic susceptibility artefact – moderate.  Rounded small subcutaneous and paravertebral signal voids | N/A | Ongoing compression by extradural IVDM and haematoma at the surgical site | Revision of surgical site | Good |
| 18 | Dachshund | 6y | FN | 593 | 4 | Extrusion | Mild | 3 | Hemilaminectomy | T12-T13 left-sided | Yes | Acute | Extradural IVDM | Mild | 1 | Present | Poorly defined bony defect, and bony hyperplasia. | Present - ipsilateral to the surgical site | Track of fatty tissue along the spinous process | Absent | Absent | Absent | N/A | IVD extrusion distant from the surgical site | Right-sided hemilaminectomy T11-T12. | Unknown |
| 19 | Basset | 7y | FE | 52 | 1 | Extrusion | Mild | 0 | Hemilaminectomy | L1-L2 left-sided | No | Peracute | Extradural IVDM | Mild | 0 | N/A^1^ | Well-defined bony defect | Present - ipsilateral to the surgical site | Focally T2W and T1W hyperintense | Absent | Absent | Metallic susceptibility artefact - moderate | 0.5 | Ongoing or recurrent extradural IVDM at the surgical site | Conservative. | Unknown |
| 20 | Lhasa Apso | 8y | FE | 10 | 180 | Protrusion | Mild | 0 | Hemilaminectomy | L4-L5 left-sided | No | Acute | Unable to assess due to metallic susceptibility artefact. | | | | | | | Present | Unable to assess due to metallic susceptibility artefact. | Metallic susceptibility artefact - marked. | 2 | Presumed to be surgical site as no other lesions were seen. | Conservative. | Euthanized due to non-neurological medical reasons. |
| 21 | French Bulldog | 4y | MN | 3 | 1 | Extrusion | Mild | 2 | Hemilaminectomy | L4-L5 right-sided | No | Acute | No compression present | Absent | 8 | Absent | Well-defined bony defect | Absent | Focally T2W hyperintense and T1W isointense | Present | Present (moderate) | Absent | N/A | Ascending myelomalacia | Euthanasia | Euthanasia |

Supplement 1: Details for individual cases.

Abbreviations: FE, Female Entire; FN, Female Neutered; MN, Male Neutered; ME, Male Entire; N/A, non-applicable; IVD, Intervertebral Disc; IVDM, Intervertebral Disc Material; T2W, T2-weighted; T1W, T1-weighted; MRI, Magnetic Resonance Imaging

^1^ For these cases, abnormal lateralization of the spinal cord within the vertebral canal was not considered as tethering due to the presence of extradural material that may have caused the lateralization.
