# Supplementary material for: Follow‐up MRI appearance of the surgical site in dogs treated for thoracolumbar intervertebral disc herniation and showing ongoing or recurrent neurological symptoms
Source: Vet Radiol Ultrasound. 2022 Aug 12;64(1):95–104. doi: 10.1111/vru.13143 (PMC10086782; doi:10.1111/vru.13143)
Supplement: Supplementary file 2 — Supplement 2: Technical parameters used for MRI studies (slice thickness, slice interval, repetition time [TR], echo time [TE], inversion recovery [IR], echo train length [ETL], and number of signals averaged [NSA]) and number of studies for each MRI sequence used. [file VRU-64-95-s002.docx]

Supplement 2: Technical parameters used for MRI studies (slice thickness, slice interval, repetition time [TR], echo time [TE], inversion recovery [IR], echo train length [ETL], and number of signals averaged [NSA]) and number of studies for each MRI sequence used.

| **Characteristics of the magnet** | **Acquisition parameters** | **T2 sag** | **T2 trans** | **T1 sag** | **T1 trans** | **T1 dorsal** | **Dorsal STIR** | **3D HYCE** |
| --- | --- | --- | --- | --- | --- | --- | --- | --- |
| 0.27 Tesla permanent MR magnet | Slice thickness (mm) | 3-4.5 | 3-5 | 3-5 | 3-5 | 3-4 | 3-4.5 | 0.35-2.5 |
|  | Slice interval  (mm) | 3.3-5 | 3.3-5 | 3-5 | 3-5 | 3.3 -4.5 | 3.5 - 5 | 0.35-2.5 |
|  | TR | 2600-5000 | 2800 - 5000 | 450 -770 | 480 - 1050 | 420 - 650 | 1020-3320 | 10 |
|  | TE | 80-120 | 80-120 | 26 | 26 | 26 | 24- 30 | 5 |
|  | IR |  |  |  |  |  | 85-90 |  |
|  | ETL | 1-8 | 1-8 | 1 | 1 | 1 | 1-4 | 1 |
|  | NSA | 1-2 | 1-2 | 1-3 | 1-3 | 1-2 | 1-2 | 1-2 |
| 1.5 Tesla MR magnet | Slice thickness (mm) | 3 | 3 | 3 | 3 |  | 4 |  |
|  | Slice interval (mm) | 3.3 | 3.3 | 3.3 | 3.3 |  | 4.4 |  |
|  | TE | 2606 | 3650 | 789 | 589 |  | 3646 |  |
|  | TE | 120 | 120 | 12 | 10 |  | 12 |  |
|  | IR |  |  |  |  |  | 160 |  |
|  | ETL | 16 | 15 | 5 | 3 |  | 9 |  |
|  | NSA | 6 | 8 | 4 | 6 |  | 2 |  |
| **Number of studies that included the specified sequence** | | 42 | 42 | 42 | 42 | 12 | 16 | 20 |

Abbreviations: mm, millimetre; MR, Magnetic Resonance; sag, sagittal; trans, transverse; STIR, Short Tau Inversion Recovery; 3D HYCE, 3-dimensional hybrid contrast enhancement.
