# Supplementary material for: Follow‐up MRI appearance of the surgical site in dogs treated for thoracolumbar intervertebral disc herniation and showing ongoing or recurrent neurological symptoms
Source: Vet Radiol Ultrasound. 2022 Aug 12;64(1):95–104. doi: 10.1111/vru.13143 (PMC10086782; doi:10.1111/vru.13143)
Supplement: Supplementary file 3 — Supplement 3: Frequencies of the main imaging findings for included dogs, according to the stage of the postoperative period at which the second MRI was performed. [file VRU-64-95-s001.docx]

Supplement 3: Frequencies of the main imaging findings for included dogs, according to the stage of the postoperative period at which the second MRI was performed.

| Assessed criteria | | Early postoperative period (0-14 days) | | | Late postoperative period (over 52 days) | |
| --- | --- | --- | --- | --- | --- | --- |
|  |  | N° cases/Total N° evaluated^1^ | % | | N° cases/Total N° evaluated^1^ | % |
| Metallic susceptibility artefacts | | 2/8 | 25% | | 5/13 | 38% |
| Extradural haemorrhage or haematoma | | 5/7 | 71% | | 0/9 | 0% |
| Extradural herniated IVDM at the surgical site | | 5/7 | 71% | | 6/9 | 67% |
| Degree of spinal cord compression at the surgical site | Marked | 1/7 | | 14% | 0/9 | 0% |
|  | Moderate | 3/7 | | 43% | 1/9 | 11% |
|  | Mild | 1/7 | | 14% | 5/9 | 56% |
| IVDH at a site distant to the surgical site | | 2/8 | 25% | | 7/13 | 54% |
| Bony defect visible with no new bone formation | | 7/7 | 100% | | 3/9 | 33% |
| Hyperplastic new bone formation | | 0/7 | 0% | | 3/9 | 33% |
| Tethering of the spinal cord to the surgical site | | 0/7 | 0% | | 4/9 | 44% |
| Increased volume and T2W hyperintense, T1W isointense epaxial muscles | | 7/7 | 100% | | 0/9 | 0% |
| Decreased volume of the epaxial muscles, normal or increased signal intensity | | 0/7 | 0% | | 5/9 | 56% |
| Collections of fluid in the paravertebral soft tissues | | 6/7 | 86% | | 0/9 | 0% |
| SC signal voids | | 5/8 | 63% | | 0/13 | 0% |
| SC T2W/T1W hypointense tract | | 8/8 | 100% | | 8/13 | 62% |
| Conservative treatment following 2^nd^ MRI | | 3/8^2^ | 38% | | 7/13 | 54% |
| Surgical treatment following 2^nd^ MRI | | 4/8^2^ | 50% | | 6/13 | 46% |

^1^ The total number of cases evaluated is 7 for the early postoperative groups and 9 for the late postoperative group wherever susceptibility artefacts impaired assessment of a specific imaging criterion.

^2^ One patient from the early postoperative period group was euthanased following a diagnosis of progressive ascending myelomalacia.

Abbreviations: SC, subcutaneous; IVDM, Intervertebral Disc Material; T2W, T2-weighted; T1W, T1-weighted; MRI, Magnetic Resonance Imaging
